# Canine Leptospirosis Outbreak in Japan

**DOI:** 10.3389/fvets.2021.763859

**Published:** 2021-12-09

**Authors:** Jun Saeki, Aki Tanaka

**Affiliations:** ^1^Department of Animal Sciences, Teikyo University of Science, Tokyo, Japan; ^2^Department of Wildlife Medicine, Nippon Veterinary and Life Science University, Tokyo, Japan

**Keywords:** leptospirosis, outbreak, Australis, dog, zoonosis

## Abstract

Canine leptospirosis was suspected in 11 dogs in Osaka Prefecture, Japan and 9 dogs died within a month, from October 12 to November 10, 2017. Eight of the dogs had been taken on walks along the same riverbed and 4 dogs lived in the same town. Logistic regression analysis between a comparative group and the incident cases group showed that the odds of leptospirosis infection was 13.3 times higher (*p* = 0.044) in the dogs taken on walks along the riverbed than in the dogs not being walked along the riverbed. It is suggesting that these walks had been a risk factor. Microscopic agglutination tests showed that antibody titers against *Leptospira interrogans* serovar Australis were 1:2,560 and 1:10,240 in 2 dogs. Therefore, *L. interrogans* serovar Australis was suspected to be the causative agent, for which no canine vaccine is available in Japan. These results suggested that *L. interrogan*s serovar Australis can cause local outbreaks. The development of a canine vaccine against various serotypes might help reduce local infections. Leptospirosis is an important infectious disease of dogs and it is also a zoonotic disease.

## Introduction

Leptospirosis is a common zoonotic disease caused by infection with the bacterium *Leptospira interrogans*, which has 24 serogroups and more than 250 known serovars (1–4). When infected with *L. interrogans*, animals may exhibit an acute course with symptoms such as jaundice, hemorrhage, and renal failure, or they might have few symptoms but continue shedding the bacteria (1–4). In some cases, animals shed the bacteria in their urine for several weeks to several years (2–4). Humans and dogs can be infected by direct or indirect contact with water and soil contaminated by the urine of wild rodents and other animals carrying the bacteria (2–4). In Japan, human infection with *L. interrogans* is categorized as a Class 4 infectious disease under the Act on the Prevention of Infectious Diseases and Medical Care for Patients with Infectious Diseases (the Infectious Diseases Control Law). In addition, the Act on Domestic Animal Infectious Diseases Control requires veterinarians to report dogs infected with *L. interrogans* serovars Pomona, Canicola, Icterohaemorrhagiae, Autumnalis, and Australis, *L. kirschneri* serovar Grippotyphosa, and *L. borgpetersenii* serovar Hardjo, as well as suspected cases. In October and November 2017, there was a series of notifications of suspected cases of canine leptospirosis based on the Act on Domestic Animal Infectious Diseases Control in Osaka Prefecture, Japan. An overview of these canine leptospirosis outbreak cases was previously reported (5). However, there have been few reports of community-acquired infections in dogs. Therefore, the aim of this study was to further analyze the outbreak and estimate the risk factors for infection.

## Methods

### Cases Investigated

The study population included dogs with suspected canine leptospirosis and dogs that visited veterinary hospitals in the northern part of Osaka Prefecture from September to November 2017 for vaccinations or other symptoms not characteristic for leptospirosis, such as skin diseases. Medical records were retrospectively evaluated and reported suspected cases of canine leptospirosis were included for analysis. Obtained data included breed, sex, age, date of onset, symptoms, basis of diagnosis, prognosis, vaccination history, dog walking route, and outing history.

An observational study design was employed to evaluate factors associated with the suspected leptospirosis.

### Statistical Analysis

The Shapiro–Wilk test was performed to evaluate the normality of the data. Logistic regression analysis was performed to evaluate the factors associated with suspected leptospirosis cases and breed, age, sex, vaccination history, type of vaccine, and history of walking along riverbeds. Stata/IC 16 (StataCorp LLC, College Station, Texas) was used for all analyses. For statistical inferences, two-sided hypothesis tests were used with a 5% significance level.

### Microscopic Agglutination Test

Sera were obtained from six cases and were tested using the microscopic agglutination test (MAT) and a panel of seven reference serotypes, as indicated by the standard method described by the International Epizootic Office (OIE) Manual of Diagnostic Tests and Vaccines for Terrestrial Animals 2021 (6). Five serovars were used: *L. interrogans* serovars Canicola (*L*. Canicola), Australis (*L*. Austlasis), Copenhageni (*L*. Copenhageni), Autumnalis (*L*. Autaumnalis), and Hebdomadis (*L*. Hebdomadis). The antibody titers for *L. interrogans* serovar Copenhageni were considered as those for *L. interrogans* serovar Icterohaemorrhagiae (*L*. Icterohaemorrhagiae) because both of them belong to the same serogroup.

## Results

### Statistical Analysis

Characteristics of the study population are presented in Table 1. The study included 19 dogs were reported by eight veterinarians in October to November 2017. Eleven dogs (case No. 4, 5, 7, 9, 10, 11, 12, 14, 16, 17, and 19) with suspected canine leptospirosis, another eight dogs (case No. 1, 2, 3, 6, 8, 13, 15, and 18) were included as a comparative group that were brought to the hospital for vaccination or other symptoms not characteristic for leptospirosis, such as skin diseases. Age ranged from 1 to 13 years, and seven Dogs were male and 12 Dogs were female. Of the 11 dogs suspected to be infected, five dogs were diagnosed as having leptospirosis based on clinical symptoms or clinical course (case No. 5, 7, 14, 16, and 17), four dogs were IgM antibody positive (case No. 9, 10, 11, and 12), and two had *Leptospira* spp. DNA detected in the blood (case No. 19) and urine (case No. 4) was analyzed by polymerase chain reaction (PCR). All six dogs that had been vaccinated with a combination of two or three *Leptospira* spp. antigens died (case No. 4, 5, 12, 14, 16, and 19), including 4 dogs that had been vaccinated within 1 year (case No. 4, 5, 14, and 19). Whereas, neither of two recovered cases had been vaccinated with *Leptospira s*pp. Antigens (case No. 7 and 11). Eight of the infected dogs lived in the same city (case No. 4, 5, 9, 11, 12, 16, 17, and 19) and four dogs lived within a 100-m radius of each other (case No. 9, 11, 12, and 17). In terms of outing history, seven of these eight dogs had been taken on walks along the same riverbed as their usual walking route (case No. 4, 5, 9, 11, 12, 16, and 19).

**Table 1:** Characteristics of the study population.

**No**.	**Reported date**	**Breed**	**Age (years)/sex**	**Address^a^**	**Onset date**	**Symptoms Purpose of visit**	**Evidence of diagnosis**	**Prognosis Condition**	**Vaccination history^b^ (year/month)**	**Walk to the riverbed**	**Death date**	**Visit date**
1	–	Toy Poodle	4 y/F	4, K-cho, J-city	–	Panniculitis	–	Good	6 combination vaccine (2014)	Yes	–	9/22
2	–	Bichon Frise	1/M	2, K-town, J-city	–	Flea parasites	–	Good	5 combination vaccine (2017)	No	–	9/26
3	–	Shiba	6/F	4, K-town, J-city	–	Combination vaccination	–	Good	5 combination vaccine (2017)	Yes	–	9/26
4	10/16	Border Collie	3/F	1, K-town, J-city	9/Late	Jaundice, DIC	IgM (–) Urine PCR (+)	Death	8 Combination vaccine (2016)	Yes	Unknown	9/late
5	10/16	Border Collie	10/M	1, K-town, J-city	9/late	Jaundice, DIC	Clinical symptoms IgM (–) Urine PCR (–)	Death	8 Combination vaccine (2016)	Yes	Unknown	9/late
6	–	Shih Tzu	9/M	1, S-town, J-city	–	Dermatitis	–	Good	Unknown	No	–	10/3
7	10/18	German Shepherd	5/M	5, M-town, L-city	10/2	Jaundice, Hepatic failure Renal failure	Clinical symptoms Urine PCR (–)	Recovery	6 Combination vaccine (2017/5)	Yes	–	10/5
8	–	Papillon	1/F	3, K-town, J-city	–	Rabies vaccination	–	Good	6 Combination vaccine (2016)	No	–	10/6
9	10/15	Mongrel	4/F	1, N-town, J-city	10/8	Severe, Acute death	IgM (+)	Death	Unvaccinated	Yes	10/11	10/11
10	10/18	Bulldog	6/F	2, Q-town, P-city	10/8	Hepatic dysfunction	IgM (+) Urine PCR (–)	Death	Unvaccinated	No	Unknown	10/12
11	10/12	Mongrel	5/F	1, N-town, J-city	10/9	Jaundice, Hepatic failure	IgM (+) Urine PCR (–)	Recovery	5 Combination vaccine (2016/9)	Yes	–	10/11
12	10/19	Mongrel	13/F	1, N-town, J-city	10/10	Pyrexia, Jaundice, Renal failure	IgM (+)	Death	9 Combination vaccine (2015/12)	Yes	Unknown	10/13
13	–	Shiba	4/F	3, K-town, J-city	–	Rabies vaccination	–	Good	Unvaccinated	Unknown	–	10/14
14	11/10	Miniature Schnauzer	8/M	2, S-town, T-ward, R-city	10/15	Severe, Acute death	Clinical progress	Death	8 Combination vaccine (2016/7)	Unknown	10/18	10/18
15	–	Miniature Dachshund	5/F	3, K-town, J-city	–	Combination vaccination	–	Good	6 Combination vaccine (2017)	No	–	10/18
16	10/29	Toy Poodle	10/M	1, K-town, J-city	10/18	Jaundice, Renal failure	Clinical symptoms Urine/Blood PCR (–)	Death	9 combination vaccine (2015/5)	Yes	10/23	10/20
17	10/26	Miniature Schnauzer	8/M	1, N-town, J-city	10/22	Hepatic failure Renal failure, thrombopenia	Clinical symptoms	Death	Unvaccinated	Unknown	10/23	10/23
18	–	Mongrel	11/F	1, N-town, J-city	-	Home visit (cohabitation dog)	–	Good	5 combination vaccine (2013)	Yes	–	10/23
19	11/9	Mix	11/F	1, R-town, J-city	11/1	Jaundice, Renal failure	Urine PCR (–) Blood PCR (+)	Death	9 combination vaccine (2016/9)	Yes	11/4	11/1

a *City and town names are not shown for privacy and anonymity*.

b *5 and 6 combination vaccines: did not contain Leptospira spp. antigens; 8 combination vaccine: including serovar Icterohaemorrhagiae and serovar Canicola antigens; 9 combination vaccine: including serovar Copenhageni, serovar Canicola, and serovar Hebdomadis antigens*.

*DIC, disseminate intravascular coagulation; F, female; M, male; PCR, polymerase chain reaction*.

There were no significant associations of vaccination within 1 year, the presence of *Leptospira* spp. antigens in the vaccine, breed, age, or sex with the occurrence of suspected symptoms of leptospirosis. The odds of leptospirosis infection were 13.3 times higher in the dogs that had a history of being taken for walks along the riverbed than in the dogs not being walked along the riverbed (*p* = 0.044).

### MAT

Antibody titers against *Leptospira s*pp. antigens in six cases are shown in Table 2. MAT was able to be performed on six cases in which the veterinarians cooperated in providing samples.

**Table 2:** Antibody titers against *Leptospira* spp. antigens in six dogs (Microscopic agglutination test).

**No**.	**Antigens**				
	**L.c**	**L.i**	**L.h**	**L.at**	**L.as**
11	<1:10	1:160	<1:10	<1:10	1:10,240
12	<1:10	1:40	<1:10	<1:10	<1:10
14	<1:10	1:320	<1:10	<1:10	<1:10
16	<1:10	1:40	<1:10	1:20	1:20
17	<1:10	<1:10	<1:10	<1:10	<1:10
19	<1;10	1:80	1:20	1:80	1:2,560

*L.c, L.Canicola; L.i, L.Icterohaemorrhagiae; L.h, L.Hebdomadis; L.at, L.Autumnalis; L.as, L.Australis*.

A titer of 1/100 is taken as a positive titer by the International Epizootic Office (OIE) Manual of Diagnostic Tests and Vaccines for Terrestrial Animals 2021 (6). The definitive diagnosis of infection was set at an antibody titers of 1:800 or higher (2, 11).

Notably, case No. 11 showed clinical signs of jaundice and hepatic failure, and the anti-*L*. Australis antibody titer was 1:10,240. Case No. 19 showed jaundice and renal failure, and the anti-*L*. Australis antibody titer was 1:2,560.

## Discussion

According to the Osaka Prefectural Livestock Hygiene Service Center, the number of canine leptospirosis cases reported in accordance with the law in Osaka Prefecture was five cases in 2014, five cases in 2015, and one case in 2016. The cases in the present study were considered to constitute an outbreak because the number of notifications was almost double that of previous years, with 11 cases reported within a month, from October 12 to November 10, 2017.

In dogs infected with *Leptospira* spp., bacteremia occurs within about 4 days of infection, and various clinical symptoms appear within about 7 days. After about 10 days of infection, antibody levels rise, and after about 2 weeks, the bacteria are excreted in the urine (4). Because the course of the disease depends on the antibody status of the individual (4), diagnoses in clinical cases should be made by selecting appropriate test methods, considering vaccination history and timing, and comprehensively evaluating clinical symptoms (2–4). In the present study, infection was confirmed in 7 cases by either MAT, PCR, or IgM antibody detection, but in three cases resulting in death due to acute progression, diagnosis was based on characteristic clinical symptoms. In another case, diagnosis was based on characteristic clinical symptoms and exclusion diagnosis.

In diagnosis by MAT, paired sera are used immediately after and 10 to 14 days after disease onset, and if the antibody titer increases more than 4-fold, infection with the suspected serovar is diagnosed (2–4). However, in many cases in the present study, progress was acute and paired sera collection was difficult, so the diagnosis was made using a single serum sample. Many cases of acute leptospirosis die within 2–4 days after disease onset, before IgG antibodies rise (2–4), and diagnosis by MAT is limited. Because there is no canine vaccine containing *L*. Australis antigens that is currently sold or used in Japan, the high antibody titers of *L*. Australis in cases No. 11 and No. 19 might indicate infection with this serovar. In a single sample, it was assumed that a high MAT titer (≥800), accompanied by clinical signs of leptospirosis, is highly suggestive of active infection (2, 11). Even in the cases where the serovar could not be identified, *Leptospira* spp. DNA was detected in the urine of case No. 4, and IgM antibody was positive in cases No. 9, 10, 11, and 12. Although PCR is a useful method for proving the presence of trace amounts of bacteria in blood, urine, and tissues, it cannot identify serovars. In an experimental case of *L*. Canicola infection, *Leptospira* spp. DNA was detected in the blood by PCR on the fourth day after infection, but not thereafter; however, it was detected in the urine after 8 days (7). Also, treatment with antibacterial agents might return a false-negative result (4). The IgM antibody test can detect antibodies against *L*. Canicola, *L*. Autaumnalis, *L*. Australis, *L*. Icterohaemorrhagiae, and *L*. Hebdomadis, but, similar to PCR, it cannot identify serovars. It is suitable as a test in the early stage of infection because IgM antibodies increase within about 3–7 days after infection before decreasing (2–4). Of the 4 dogs that tested positive for IgM antibodies, cases No. 9, 11 and 12 lived in the same town and developed the disease over 3 days from October 8 to 10. This finding suggests that they might have been infected at about the same time by the same source. Of the 11 dogs with suspected infection, eight dogs lived in the same city and neighboring cities. Furthermore, seven of these eight dogs were taken on walks along the same riverbed as their usual walking route, and epidemiological analysis indicated that they were infected at this site. Recently, there has been an increase in the number of feral raccoons (*Procyon lotor*) in Japan because of abandonment of pet raccoons imported to Japan from North America (8). These animals have been implicated as a source of zoonotic pathogens, including *Leptospira* spp. (9). Raccoons living in the vicinity of the outbreak area are also known to have high antibody titers against *Leptospira* spp. (10) and are considered a possible source of infection. There have been few reports of outbreaks of infectious diseases in household dogs. Often the number of cases is also small, so conducting epidemiological studies is difficult. In the present study, factors associated with an outbreak of canine leptospirosis were evaluated.

*L*. Hebdomadis has been documented as the most common cause of canine leptospirosis in Japan, accounting for 53.3% of all reported cases (11). Although vaccination is reported to be effective in preventing onset and reducing the severity of canine leptospirosis (12), effectiveness is considered serovar-specific (13). Currently, the *Leptospira* spp. canine vaccine available in Japan contains only *L*. Canicola, *L*. Icterohaemorrhagiae, *L*. Hebdomadis, *L. interrogans* serovars Pomona and *L. kirschneri* serovar Grippotyphosa antigens, and does not contain the antigens of *L*. Australis detected in the present study. This might explain why some dogs died in this outbreak despite being vaccinated against *Leptospira* spp. There are known to be regional differences in the detected serovars (3, 14, 15). Because *L*. Australis has been reported to be the second-highest detected serovar (20.3%) after *L*. Hebdomadis in dogs in Japan (14), it is necessary to develop a vaccine containing antigens of this serovar. It is also desirable to develop vaccines in other countries and regions according to the outbreak situation.

### Limitation of Study

Because this was a field survey of spontaneous cases, the number of cases was small and most of the dogs were not available for a long term follow up, some of the dogs died during the study period due to the illness. In addition, we were not able to obtain the complete information on the cases we evaluated.

## Data Availability Statement

The original contributions presented in the study are included in the article/supplementary material, further inquiries can be directed to the corresponding author.

## Author Contributions

JS was involved in the study design and data interpretation. AT was involved in the data analysis. Both authors critically revised the report, commented on drafts of the manuscript, and approved the final report.

## Conflict of Interest

The authors declare that the research was conducted in the absence of any commercial or financial relationships that could be construed as a potential conflict of interest.

## Publisher's Note

All claims expressed in this article are solely those of the authors and do not necessarily represent those of their affiliated organizations, or those of the publisher, the editors and the reviewers. Any product that may be evaluated in this article, or claim that may be made by its manufacturer, is not guaranteed or endorsed by the publisher.
